# Good vs complementary genes for parasite resistance and the evolution of mate choice

**DOI:** 10.1186/1471-2148-4-48

**Published:** 2004-11-19

**Authors:** R Stephen Howard, Curtis M Lively

**Affiliations:** 1 Department of Biology, Middle Tennessee State University, Murfreesboro, TN 37132, USA; 2 Department of Biology, Indiana University, Bloomington, IN 47405-3700, USA

## Abstract

**Background:**

Female mate choice may be adaptive when males exhibit heritable genetic variation at loci encoding resistance to infectious disease. The Hamilton-Zuk hypothesis predicts that females should assess the genetic quality of males by monitoring traits that indicate health and vigor (condition-dependent choice, or CD). Alternatively, some females may employ a more direct method of screening and select mates based on the dissimilarity of alleles at the major histocompatibility loci (we refer to this as opposites-attract, or OA). Empirical studies suggest that both forms of mate choice exist, but little is known about the potential for natural selection to shape the two strategies in nature.

**Results:**

We used computer simulation models to examine the evolutionary fates of the two forms of mate choice in populations at risk for infection by debilitating parasites. We found that populations exhibiting random mating (no mate choice) can be invaded and replaced completely by individuals practicing CD type mate choice. We also found that an allele encoding OA choice can increase when rare in randomly mating populations, but that it does not go to fixation under selection. A similar result was obtained when the OA strategy was introduced into populations practicing CD mate choice. As before, we found that the OA choice allele will increase when rare, and that it will not go to fixation under selection. The converse however was not true, as CD individuals gain no rare advantage when introduced into an OA population.

**Conclusions:**

Taken together, the results suggest that, when rare, OA is the best strategy for parasite evasion (of those considered here). The consequence of OA increasing in the population, however, is to reduce the parasite driven genotype oscillations and facilitate the breakdown of linkage disequilibrium at the disease-resistance loci. This leads to a neutrally stable situation in which different strategies have equal fitness, and suggests that multiple forms of mate choice may be expected to occur in populations at risk from infectious disease.

## Background

Since Darwin published his classic work on sexual selection [1], biologists have puzzled over the evolutionary consequences of mate choice. As a general rule, evolutionary theory predicts that females rather than males will benefit most from discriminating among potential mates [2]. A large body of evidence now supports the hypothesis that female mate choice is a potent evolutionary force, but its causes and consequences can vary widely. For example, choosy females may benefit directly through the acquisition of valuable resources [3], may gain indirect benefits by obtaining better genes for their offspring [4], or may simply prefer mates due to some preexisting sensory bias [5,6]. In the early 1980's, Hamilton and Zuk hypothesized that females discriminate in favor of males bearing genotypes encoding resistance to coevolved parasites [7]. A key prediction of this model is that females should select males based on indicator traits that reveal the bearer's status of infection. Investigations of mate choice in birds and fish support the prediction that females may indirectly discriminate against parasitized males by selecting brightly colored males [8-12], but other studies suggest that females may use cues other than condition to identify males with genes that could contribute to disease resistance. For example, results from recent experiments suggest that females of some species select males based on their genetic configuration at the major histocompatibility complex (MHC) loci. The role of the MHC in the vertebrate immune response is well established, and is known to enhance resistance to many species of endoparasites [13,14]. Hence, it would seem that females could benefit from mating preferentially with males having favorable MHC profiles, provided they can identify these males in the population. This seems plausible, as recent studies have documented its occurrence in mice and rats [15-17], humans [18,19], and fish [20-23].

In a previous study, we investigated the potential for active mate choice to indirectly favor the maintenance of sexual reproduction [24]. We found that sexual populations in which females prefer to mate with males having dissimilar resistance alleles (which we called OA for opposites attract) are more resistant to invasion by parthenogenetic clones than those in which females prefer uninfected males (a condition dependent strategy, CD). In the present study, we investigate the potential for OA and CD mate choice to generate a selective advantage to females at the level of the individual, independent of the incidental effects that such choice may have on the evolutionary stability of sex.

## Results

**Figure 1:**
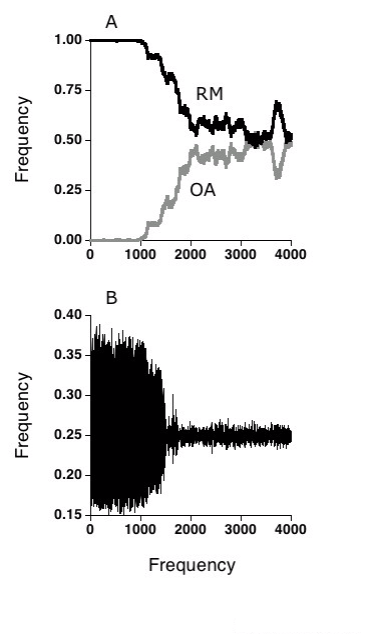
Results from a single run of a two-locus, two-allele version of the simulation in which a rare mutant for the opposites attract (OA) strategy was introduced into a randomly mating (RM) population of 20,000 hosts. At generation 200, the mutation rate between OA and RM was set at 0.000001 per individual per generation. **(a) **Once established, the OA allele spread until its frequency was about 0.5 in the population. **(b) **The 1,1 host genotype frequency at the two interaction loci before and after the invasion of the OA choice allele. Note the drastic reduction in the amplitude of the oscillations which accompany the spread of the OA allele.

**Figure 2:**
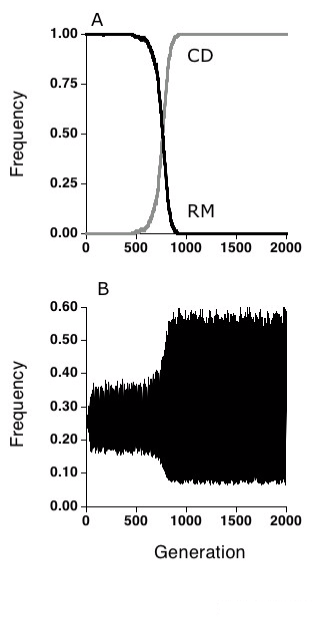
Results from a single run of a two-locus, two-allele version of the simulation in which a rare mutant for the condition dependent (CD) strategy was introduced into a randomly mating population of 20,000 individuals. At generation 200, the mutation rate between CD and RM was set at 0.000001 per individual per generation. **(a) **Once established, the CD allele spread until fixation. **(b) **The 1,1 host genotype frequency at the two interaction loci before and after the invasion of the OA choice allele. Note the increase in amplitude of the oscillations which accompany the spread of the CD allele.

The results from our study show that when populations are confronted with infectious disease, alleles encoding OA and CD mate choice both increase when rare in randomly mating populations (Figs. 1A and 2A). Hence the results are consistent with the basic idea that mate choice will be spread in populations engaged in coevolutionary interactions with parasites. However, the two different kinds of mate choice had different effects on the population genetic dynamics for multilocus genotypes associated with disease resistance.

### OA-choice vs. random mating

As the allele for OA increased in the population, the oscillations in both host and parasite resistance genotypes underwent a dramatic reduction in amplitude (Fig 1B). In addition, as the oscillations became damped, the spread of the OA allele into the population was slowed. This result makes sense, as rare resistance genotypes have an advantage when OA enters the population, whether or not they are over or under infected (Note that over infection of rare types occurs during part of the coevolutionary cycle). However, the advantage under sexual selection is not lagged in time (as is the advantage due to parasite resistance), and the amplitude of the oscillations becomes damped as the OA phenotype increases in the population. As the oscillations become damped and the genotypes become equally frequent (barring some drift), the value of mate choice is diminished, and the spread of the OA allele is halted. At this point, OA and RM are selectively neutral, and their frequencies in the population change only as the result of drift. Selection for OA, however, is reestablished if the OA allele drifts below 0.5, as this reestablishes conditions favoring the production of rare resistance genotypes.

### CD-choice vs. random mating

In contrast, as the allele for CD choice increased from its rare starting point, the oscillations in genotype frequency increased, rather than decreased (Fig. 2B). This result also makes sense, as genotypes that are least infected are also more likely to be selected as mates, and both advantages are lagged in time. This increases the selection for genotypes that are currently underinfected, and increasing the selection differential increases the amplitude of the oscillations. It also greatly increases the oscillations in linkage disequilibrium over time [24]. In addition, unlike the OA allele, the CD allele goes to fixation in an RM population. Thus it would appear that in the presence of parasites, there is sustained selection against random mating, even when CD-choice is common.

### OA-choice vs. CD-choice

**Figure 3:**
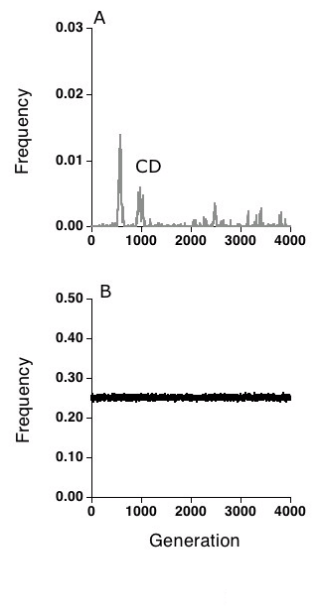
Results from a two-locus, two-allele version of the simulation in which a rare mutant for CD mate choice was introduced into an OA population of 20,000 individuals. At generation 200, the mutation rate between CD and OA was set at 0.000001 per individual per generation. **(a)**. The CD allele failed to become established over the course of 4,000 generations **(b) **Frequency of the 1,1 host genotype.

Under the conditions studied here, both OA and CD alleles increase when rare in randomly mating populations, but only the CD allele goes to fixation due to sexual selection. How does CD fare against an OA population? We found that when introduced into a population of 20,000 OA individuals, a rare allele encoding CD choice was not favored by selection, but that it could become established and increase in frequency as a result of genetic drift (Fig. 3). This result appears to stem from the fact that in a population dominated by OA individuals, the disease-resistance genotypes are virtually equally frequent and equally infected; hence there is little selective value in choosing the least infected individuals as mates. Thus OA seems to be an evolutionary stable strategy (ESS).

**Figure 4:**
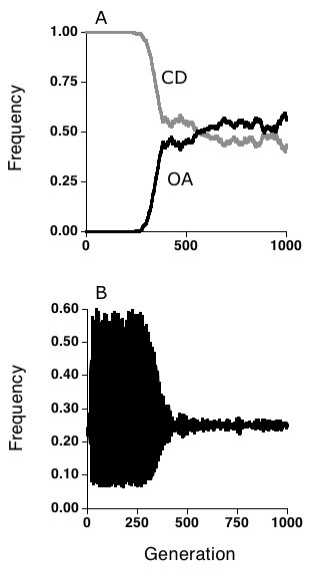
Results from a two-locus, two-allele run of the simulation in which a rare mutant for OA mate choice was introduced into a CD population of 20,000 individuals. At host generation 200, the mutation rate between CD and OA was set at 0.000001 per individual per generation. **(a)**. Once established, the OA allele spread rapidly until it reached a frequency of about 0.5 in the population. **(b) **Frequency of the 1,1 host genotype. Note the rapid attenuation of cycling which accompanied the spread of the OA allele into the population.

**Figure 5:**
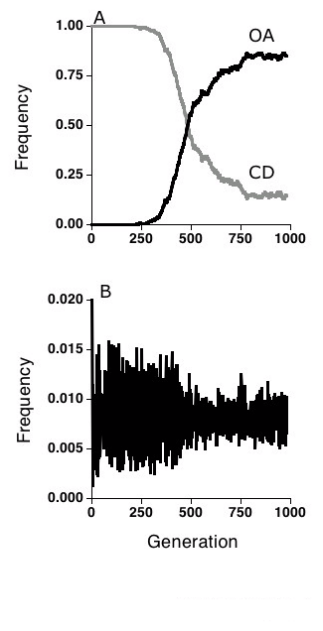
Results from a three-locus, five-allele run of the simulation in which a rare mutant for OA mate choice was introduced into a CD population of 20,000 individuals. At generation 200, the mutation rate between CD and OA was set at 0.000001 per individual per generation. **(a)**. Once established, the OA allele spread rapidly until it reached a frequency of about 0.8 in the population. **(b) **Frequency of the 1,1,1 host genotype. Note that in this version of the simulation there are a possible one hundred and twenty five host and parasite interaction genotypes.

In contrast, OA did increase when rare due to selection in a population of CD individuals; hence CD is not an ESS (Fig. 4). The overall pattern was similar to that obtained from our previous runs of OA against a RM population. As before, the oscillations in genotype frequencies became damped as OA increased in the population, and the OA allele lost its selective advantage and began to drift. The result is apparently for the same reasons as given above: as OA spreads the oscillations become damped and the resistance genotypes become equally frequent and equally infected. At this point CD and OA are selectively neutral. Interestingly, the extent to which OA spread under selection appeared to increase in the three-locus, five-allele version of the simulation (Fig. 5).

## Conclusions

Among the three strategies of mate choice for parasite avoidance considered in this study, only OA is evolutionary stable. However, OA will not eliminate either RM or CD-choice through the action of sexual selection, since OA becomes selectively neutral at intermediate frequencies in both situations by causing reductions in the fitness variance among resistance genotypes. Thus, OA and RM, OA and CD, or all three could coexist in the short term in large populations (until OA goes to fixation by drift). As such, in populations at risk from disease, females might practice more than a single type of mate choice.

## Methods

The methods employed in this investigation are similar to those used in our previous studies of host parasite coevolution [24,25]. We used individual-based computer simulation models to track the dynamics of alleles encoding different strategies of mate choice in host populations at risk of infection by debilitating parasites. Both hosts and parasites were modeled as sexual hermaphrodites with discrete, non-overlapping generations; parasites underwent two generations for each host generation. The antagonistic interaction was mediated by a matching alleles model of infection in which successful parasites were required to match hosts exactly at each of two or three freely-recombining loci (interaction loci). In the simplest form of the model, the interaction was mediated by two alleles at each of two loci, which allowed for a total of four possible resistance genotypes. We also conducted runs for a three-locus version of the model, with five resistance alleles at each locus. This configuration allowed for the production of one hundred and twenty five genotypes. The mutation rate between the alternative alleles at each of the interaction loci was set at 0.03 per generation in the parasite population. This was done to prevent the fixation of parasite alleles under the conditions studied, e.g. where high risks of parasite exposure are coupled with moderate to high levels of virulence.

During each parasite generation, hosts were drawn sequentially and exposed to a randomly drawn parasite with a probability of *T*. For the two locus version of the model, T was set to a value of 0.8, and parasites underwent two generations for each host generation. In the three-locus, five-allele version of the model, higher rates of parasite transmission coupled with a greater asymmetry of host and parasite generation times were required to sustain infection in the host population. For this version of the model, T was set to a Poisson distributed mean of eight, and parasites underwent ten generations for each host generation. In all cases, if a parasite matched a host exactly at all interaction loci, the host was marked as infected and the parasite was placed in a pool of potential reproductives. Once infected, individual hosts were protected against further attack by parasites. Reproduction in hosts and parasites was accomplished by drawing individuals from their respective populations at random with replacement. The parasite life cycle included a "free-living" stage in which reproductive adults emerged from infected hosts to mate. When an individual (host or parasite) was selected for reproduction, a second individual was randomly selected for cross-fertilization. Gametes from the two haploid "parents" were then brought together to form a diploid zygote stage, where free recombination between the interaction loci made possible the production of a genetically diverse brood of haploid offspring. All else equal, sexual individuals (hosts and parasites) produced a lifetime average of 10 haploid offspring. The number of offspring produced by hosts, however, was reduced according to the status of parasitic infection for each of the parents. The reproductive output of each host parent was discounted according to 10(1-*E*), where *E *simulates the detrimental effect of parasitism on host reproduction (virulence). In the present study, *E *was set to a value of 0.8 to simulate the effect of a moderately virulent parasite. Following reproduction, a maximum of 20,000 parasite and 20,000 host offspring were selected at random to become the next generation of adults.

At the beginning of each run, the interaction alleles at each locus in the host and parasite populations were initialized to a frequency of 1/*n*, where *n *was the total number of alleles present at each locus. Prior to data collection, the simulation was allowed to run for 200 host generations; this allowed for establishment of long-term coevolutionary dynamics arising from the host-parasite interaction. Next, we tracked the fates of alleles encoding different types of mate choice in the host population. Mating preferences in hosts were controlled by a single locus encoding one of three strategies: random mating (RM), condition-dependent mate choice (CD), or opposites attract (OA) mate choice. Random mating was simulated by pairing two randomly drawn adults. Condition-dependent choice was implemented by allowing the first reproductive drawn (the "female") to identify and discriminate against parasitized "males". For the opposites attract strategy, females preferred "males" with alleles different from their own at each of the two interaction loci. For both OA and CD choice, "females" were allowed to choose the "best" available male from a sequence of 20 randomly drawn "males".

## Authors' contributions

RSH wrote the computer code and implemented the simulations. Both authors participated equally in all other phases of the work.
